# Fusion of Technology in Cochlear Implantation Surgery: Investigation of Fluoroscopically Assisted Robotic Electrode Insertion

**DOI:** 10.3389/fsurg.2021.741401

**Published:** 2021-11-08

**Authors:** Greg Eigner Jablonski, Benedicte Falkenberg-Jensen, Marie Bunne, Muneera Iftikhar, Ralf Greisiger, Leif Runar Opheim, Hilde Korslund, Marte Myhrum, Torquil Mcdonald Sørensen

**Affiliations:** ^1^Institute of Clinical Medicine, University of Oslo, Oslo, Norway; ^2^Department of Otorhinolaryngology & Head and Neck Surgery, Oslo University Hospital, Rikshospitalet, Oslo, Norway; ^3^Department of Radiology, Oslo University Hospital, Rikshospitalet, Oslo, Norway; ^4^Interventional Centre, Oslo University Hospital, Rikshospitalet, Oslo, Norway

**Keywords:** cochlear implant, robot-assisted cochlear implantation, RCI, HEARO®, robot-assisted surgery, OTOPLAN®, fluoroscopy at implantation, CBCT (cone beam computed tomography)

## Abstract

The HEARO cochlear implantation surgery aims to replace the conventional wide mastoidectomy approach with a minimally invasive direct cochlear access. The main advantage of the HEARO access would be that the trajectory accommodates the optimal and individualized insertion parameters such as type of cochlear access and trajectory angles into the cochlea. To investigate the quality of electrode insertion with the HEARO procedure, the insertion process was inspected under fluoroscopy in 16 human cadaver temporal bones. Prior to the insertion, the robotic middle and inner ear access were performed through the HEARO procedures. The status of the insertion was analyzed on the post-operative image with Siemens Artis Pheno (Siemens AG, Munich, Germany). The completion of the full HEARO procedure, including the robotic inner ear access and fluoroscopy electrode insertion, was possible in all 16 cases. It was possible to insert the electrode in all 16 cases through the drilled tunnel. However, one case in which the full cochlea was not visible on the post-operative image for analysis was excluded. The post-operative analysis of the electrode insertion showed an average insertion angle of 507°, which is equivalent to 1.4 turns of the cochlea, and minimal and maximal insertion angles were recorded as 373° (1 cochlear turn) and 645° (1.8 cochlear turn), respectively. The fluoroscopy inspection indicated no sign of complications during the insertion.

## Introduction

Cochlear implantation has been the gold standard treatment for severe to profound sensorineural hearing loss over several decades (1). This conventional surgical method is well established and practiced in many countries; however, its success relies not only on the surgical skills but also on the anatomical variations in the patient. To overcome these variables, the development of robotic cochlear implantation took place.

The idea behind robotic cochlear implantation is to obtain a system that is minimally invasive, reproducible, reliable, safe, and effective. Robotic cochlear implantation is an image-guided system, which drills a trajectory from the mastoid surface to the middle ear bypassing the critical anatomical structures such as the facial nerve, chorda tympani, ossicles, and the posterior ear canal wall. Caversaccio (2), Labadie (3), and Bell (4) have previously described the safety and success of this procedure; however, their experience with robotic assisted surgery was limited to the middle ear access and not to the inner ear.

Our aim is to describe further development of this method by using the robotic cochlear implantation to access the round window (RW), without damaging the critical structures and achieve an optimized angle for manual electrode insertion through the drilled trajectory.

This is achieved by using OTOPLAN, which is an image-based system that allows accurate patient-to-image registration using bone anchored fiducial screws implanted into the mastoid surface. The system calculates a distance to the facial nerve, chorda tympani, the ossicles, the posterior ear canal wall, and plans out a trajectory from the mastoid surface to the RW, as described by Weber et al. (5).

Such a procedure does not only demand thorough radiological planning but also visual inspection and assessment of repetitive achievement of RW access to the cochlea. We have utilized transtympanal endoscopy (0-degree endoscope, Karl Storz) together with fluoroscopy and Cone Beam CT (CBCT) acquired by Siemens Artis Pheno with robotic C-arm (Siemens AG, Germany). This was possible due to the advanced fusion of technology at our Intervention Centre, Oslo University Hospital, Norway.

There has been a major development in robotically assisted cochlear implantation surgery over the last decade. The image-guided robot system has been shown to be highly precise and safe as previously described by Bell (4), Caversaccio (2), and Weber (5). Robotically assisted cochlear implantation has been performed by drilling a direct tunnel from the mastoid surface to the middle ear and gaining manual access to the inner ear, keyhole access (2, 6). Considering microanatomy with the closely adjacent facial nerve and chorda tympani, the procedure requires high levels of navigation accuracy and additional independent tool position and orientation methodologies (7). The image-guided robotic system described by Caversaccio (2) and Weber (5) has demonstrated a high level of tool positioning accuracy and precision (0.15 ± 0.08 mm at the level of cochlea).

In a procedure like robotic cochlear implantation, which is performed at a microsurgical scale with submillimetric distance to the facial nerve, the necessity for several safety mechanisms is paramount–in case of navigation error and to avoid thermal and mechanical damage to the relevant anatomical structures.

It utilizes (a) Visual surveying scheme by an optical position measurement system that tracks the end effector of the robot and the head of the patient by means of rigidly fixed optical reference, (b) estimates of the drill position and orientation by correlating the drill force and bone density, (c) neuro-stimulation feedback mechanism of the facial nerve and interval drilling with saline flushing during the interval to minimize heat accumulation, and (d) intra-operative CBCT imaging (Siemens Artis Pheno). Previous studies have demonstrated the reliability of robotic cochlear implantation where the trajectory has been drilled from the mastoid surface to the middle ear and thereby gaining manual access to the cochlea for electrode placement (2, 5–7).

The aim of our study was to gain access to the RW solely by the image-guided robotic system rather than manually drilling into the RW.

The surgeon controls the robotic drilling by continuously pressing a pedal. Hence, the drilling can be stopped immediately at any time by releasing the pedal.

## Materials and Methods

The Regional Committee for Medical and Health Research Ethics of Northern Norway evaluated the *ex vivo* study (REC North, reference: 2018/378/REK nord). Eight formalin-flushed (C7-Th1) *ex vivo* human head specimens (16 temporal bones) were included in this study. The HEARO procedure workflow included the following steps:

1. Incision and fiducial screw placement
2. Imaging and planning
3. Robotic middle ear access
4. Robotic inner ear access
5. Electrode insertion
6. Post-operative analysis.

### Incision and Screw Placement

The Robotic arm was attached to the operating room (OR) table and covered in sterile draping. The human cadaver head specimen was placed relative to the robot system and immobilized in the radio-translucent HEARO headrest, using inflatable pressure pads. A C-shaped retroauricular incision was performed on all cases, and a retractor was used to keep the skin flaps away from the surgical field. After incision, five fiducial screws (four for patient-to-image registration and one for assembling of the patient marker attachment) were inserted into the surface of the mastoid (8).

### Imaging and Planning

After the fiducial screws were positioned, a high-resolution CBCT image was acquired by Siemens Artis Pheno with Robotic C-arm (Siemens AG, Germany). A 0.1 mm reconstruction protocol was used for the surgical planning. The image quality was validated by excluding artifacts and confirming the inclusion of all four registration screws, facial canal, middle ear, and labyrinth (9). The raw data were then transferred to OTOPLAN software (CASCINATION AG, Switzerland). A senior radiologist identified, and 3D reconstructed the following structures using a semi-automatic segmentation algorithm:

- External ear canal
- The ossicles including the stapes
- The RW of the cochlea
- The tympanic and mastoid segment of the facial nerve
- The mastoid segment of the chorda tympani.

The target point of the drilling was placed on the RW. The OTOPLAN software automatically calculates distances from the drilling tunnel to the surrounding anatomy. This trajectory was adjusted to a minimum safe distance of 0.4 mm to the facial nerve, and 0.3 mm to the external auditory canal, ossicles, and the chorda tympani. Before exporting the planned trajectory to HEARO, a qualified senior ear surgeon approved the trajectory.

### Robotic Middle Ear Access

After the patient-to-image registration, the first stage of drilling was carried out from the mastoid surface to 3 mm from the level of the facial nerve. The keyhole was created using a drill bit with a diameter of 1.8 mm. Interval drilling and irrigation were carried out with a drilling speed of 1,000 rpm. On completion of the first stage of drilling, the patient marker was removed, and a trajectory reference rod was fitted into the tunnel. An intra-operative CBCT image (Siemens Artis Pheno) was performed to assess the safety and accuracy of the drilling trajectory. On confirmation, the patient marker was assembled and the registration was repeated. The second stage of the drilling was commenced with a reduced drilling interval depth of 0.5 mm, in contrast to the 2 mm of the first stage (8). The second stage of drilling stopped at the drill stopping point prior to the RW according to the plan from OTOPLAN.

An additional tympanomeatal flap was created for direct endoscopic visualization of the tympanic cavity, and manual assistance of electrode array insertion procedure. The alignment of the drilled tunnel with the target (RW) was visually confirmed with a microscope and/or 0-degree endoscope (Karl Storz, diameter of 2.7 mm, Germany) through the external auditory canal into the middle ear while inserting a titanium rod through the drilled tunnel.

### Robotic Inner Ear Access

The milling of the inner ear access was carried out with a 1 mm diamond burr at a feed rate of 0.01 mm/s, 2,000 rpm. The milling was performed under direct visualization using a 0-degree endoscope (Karl Storz, Germany) and/or microscope. Water irrigation was not used for milling. The surgeon actively terminated milling by releasing the pedal, when the force sensitive measurement (10) on the screen showed a sudden drop, indicating a sufficient opening through the bony overhang of the RW. Finally, the surgeon performed a visual evaluation of the RW membrane integrity.

### Electrode Insertion

Prior to electrode insertion, the drilled tunnel was cleansed of bone dust. A protective barrier guide tube was used to prevent any kinking or displacement of the electrode into the air cells during insertion. We used a radio-translucent biodegradable-type tube, which could be left inside the channel after insertion in nine cases, and a two-part longitudinally divided metallic version in seven cases, which had to be removed after electrode insertion. Prior to the insertion, the RW membrane was removed manually using a micro-needle. The electrode array was coated with hyaluronic acid [12 mg/ml, stabilized, Restylane Skinboosters (Vital &Vital Light)] to minimize friction, and then inserted manually under visual (with endoscope/microscope) and fluoroscopic guidance. Fluoroscopic monitoring was performed using a CBCT (Siemens Artis Pheno). The cadaver head was placed in a carbon fiber HEARO headrest to avoid any artifact. The X-ray source was placed under the operating table and the detector above the cadaver head specimen, ~90-degree angle of the X-ray direction to the plane of the basal turn of the cochlea. During fluoroscopy, an X-ray tube acceleration voltage of ~70 kV and a tube current of ~250 mA were used. The fluoroscopy frame rate was set to four or five frames per second. A FLEX28 electrode (MED-EL, Innsbruck, Austria) was used in all cases for the insertion. The electrode array contacts and internal wiring were visible on the fluoroscopy, despite the presence of the guide tube. We did not perform sealing around the electrode, as there was not much space left around the electrode after insertion.

### Post-operative Analysis

Upon completion of the electrode insertion, a post-operative CBCT scan was performed (also using the Siemens Artis Pheno, and a cubic voxel size of side length 0.1 mm was used in the volume reconstruction) in order to analyze the insertion status of the electrode using the post-operative analysis feature of OTOPLAN.

## Results

**Figure 1:**
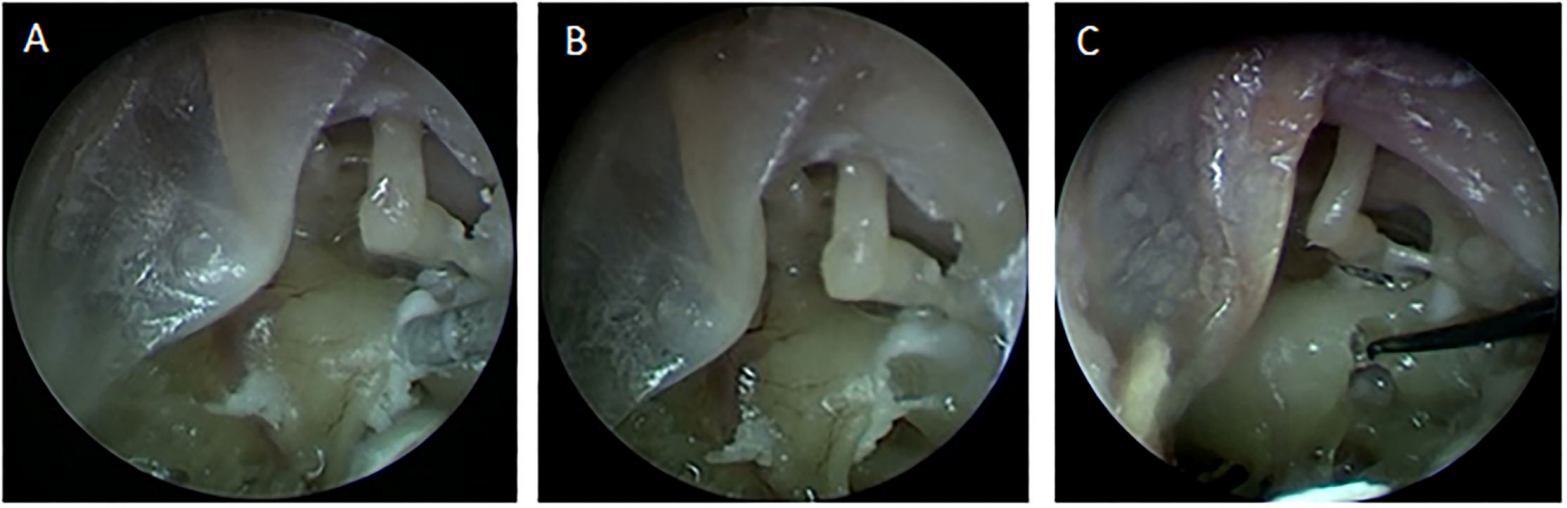
**(A)** The milling of the inner ear access with visual endoscopic inspection through the tympanomeatal flap. **(B)** The 1 mm inner ear access created for the electrode insertion. **(C)** Opening of the Round Window membrane using a micro-needle.

The completion of the full HEARO procedure, including the robotic inner ear access and the intra-operative analysis, has shown no damage to the facial nerve during the middle ear access. Intra-operative and post-operative CBCT showed and maintained visible bone borders between the drilled canal and the facial nerve, the chorda tympani, and the external ear canal in all cases. In addition, there was no damage to the ossicles. The visual endoscopic supervision of creating a 1 mm window on the bony overhang of the cochlea and subsequent opening of the RW membrane is shown in Figure 1.

**Figure 2:**
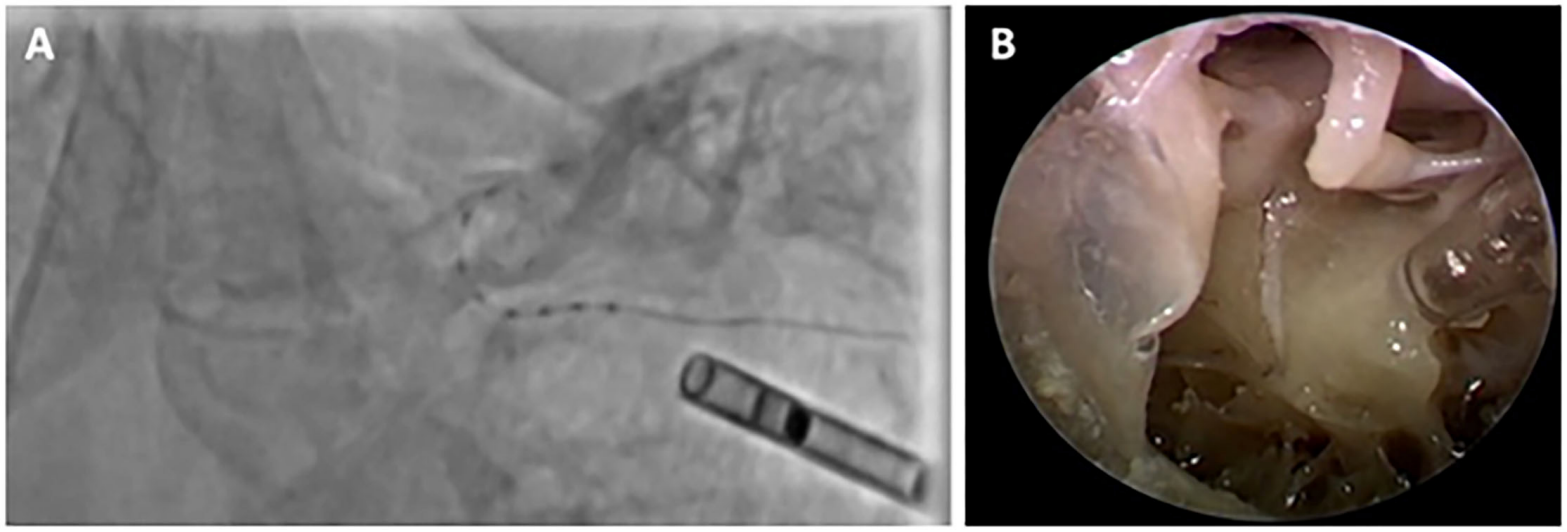
**(A)** The electrode insertion through fluoroscopic view, **(B)** Electrode insertion through the transparent tube, endoscopically supervised.

It was possible to insert the electrode in all 16 cases through the drilled tunnel after the robotic inner ear access under the fluoroscopy guidance. A protective barrier tube was placed inside the tunnel prior to the insertion. An example of fluoroscopy and endoscopic supervision of the electrode insertion process is shown in Figure 2.

**Figure 3:**
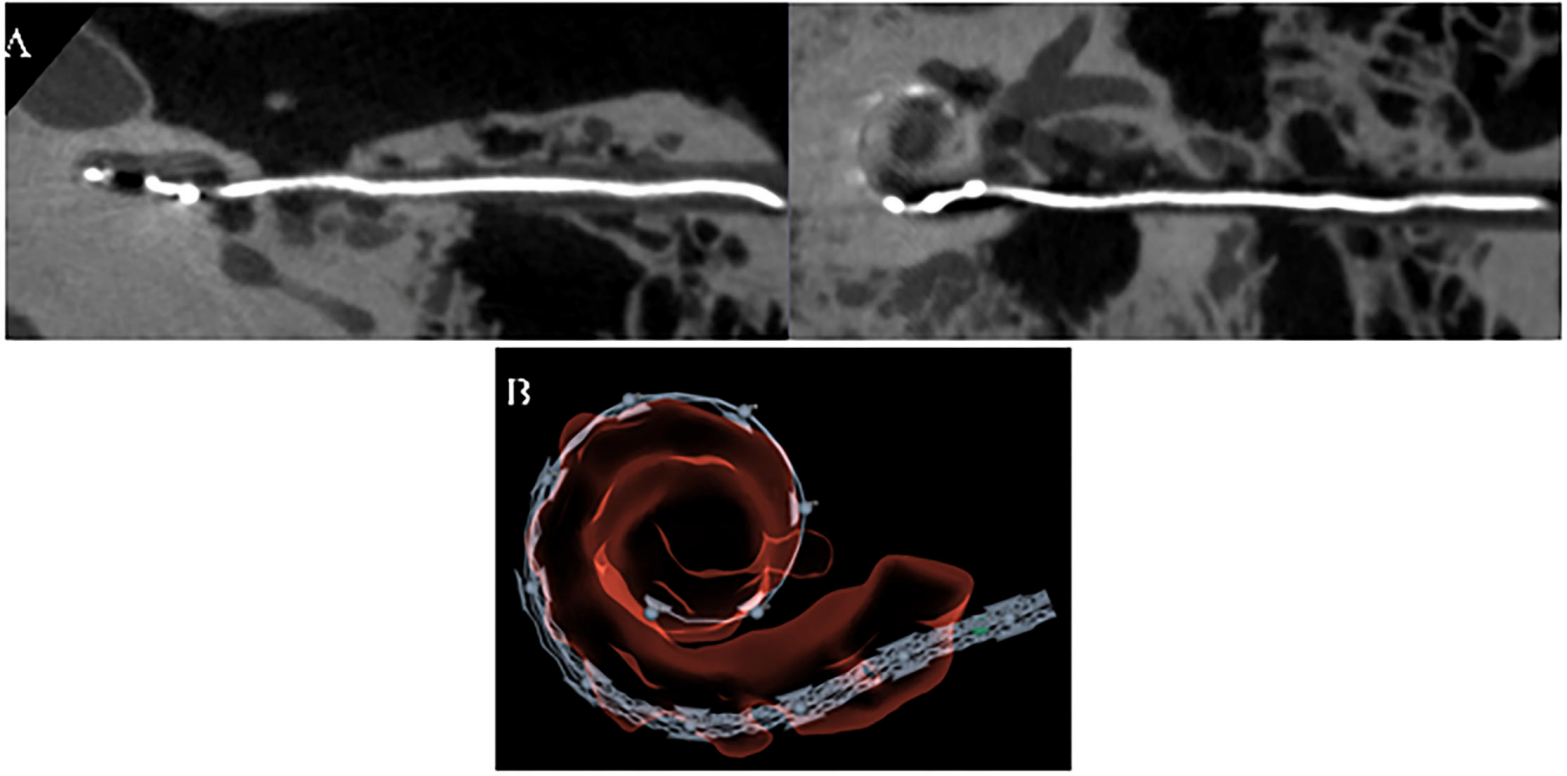
Post-op analysis of electrode insertion using **(A)**. CBCT Siemens Artis Pheno and **(B)**. OTOPLAN®.

Post-operatively the insertion status of all cases was verified with CBCT (Siemens Artis Pheno) and analyzed using the OTOPLAN software to determine the insertion depth, electrode location, and possibility of tip fold-over or scala deviation. An example of such analysis is shown in Figure 3.

One case (03_Left), in which the full cochlea was not visible on the post-operative image for analysis, was excluded. The post-operative analysis of the electrode insertion showed a full insertion in 12 cases. A minimum of one turn of the cochlea was covered in all cases. The average insertion angle in 15 cases was 507°, which is equivalent to 1.4 turns of the cochlea, and minimum and maximum insertion angles were recorded as 373° (1 cochlear turn) and 645° (1.8 cochlear turns), respectively. The partial insertion cases left a maximum of two contacts out, possibly due to the formalin fixation of the specimen. No tip fold-over or scala deviation was observed in the 15 cases. The summary and details of the insertion status of the post-operative images are shown in Tables 1, 2, respectively.

**Table 1:** Insertion status analysis from post-operative images.

**Description**	**Number of cases (*n* = 15)**
Full insertion	12/15
Average insertion angle	507° (1.4 Turns)
Min insertion angle	373° (1 Turn)
Max insertion angle	645° (1.8 Turns)
Tip fold-over	0/15
Scala deviation	0/15

**Table 2:** Detailed insertion status analysis from post-operative images.

**Cases**	**Cochlea parameters**	**Insertion status**
00_Left	Diameter (A) = 8.9 mm Width (B) = 7.1 mm Height (H) =3.6 mm CDL (from RW) = 34.1 mm	Insertion depth: 543° Full insertion Location: Scala tympani Scala deviation: No Tip fold-over: No
00_Right	Diameter (A) = 9.1 mm Width (B) = 6.7 mm Height (H) =3.3 mm CDL (from RW) = 32.8 mm	Insertion depth: 508° Full insertion Location: Scala tympani Scala deviation: No Tip fold-over: No
01_Left	Diameter (A) = 9.2 mm Width (B) =6.7 mm Height (H) =3.4 CDL (from RW) = 33.2mm	Insertion depth: 476° Full insertion Location: Scala tympani Scala deviation: No Tip fold-over: No
01_Right	Diameter (A)= 9.1 mm Width (B) = 6.5 mm Height (H) =4.0 mm CDL (from RW) = 32.0 mm	Insertion depth: 426° Full insertion Location: Scala tympani Scala deviation: No Tip fold-over: No
02_Left	Diameter (A) = 9.1 mm Width (B) =6.5 mm Height (H) =3.7 mm CDL (from RW) = 32.2 mm	Insertion depth: 586° Full insertion Location: Scala tympani Scala deviation: No Tip fold-over: No
02_Right	Diameter (A) = 9.2 mm Width (B) =6.5 mm Height (H) =3.5 mm CDL (from RW) = 32.1 mm	Insertion depth: 521° Full insertion Location: Scala tympani Scala deviation: No
03_Left	Skipped (image is cut)	/
03_Right	Diameter (A) = 8.7 mm Width (B) = 6.3 mm Height (H) = 3.8 mm CDL (from RW) = 31.0 mm	Insertion depth: 517° Full insertion Location: Scala tympani Scala deviation: No Tip fold-over: No
04_Left	Diameter (A) = 9.4 mm Width (B) =6.3 mm Height (H) =3.7 mm CDL (from RW) = 31.9 mm	Insertion depth: 557° Full insertion Location: Scala tympani Scala deviation: No Tip fold-over: No
04_Right	Diameter (A) = 9.3 mm Width (B) = 6.6 mm Height (H) = 3.4 mm CDL (from RW) = 32.7 mm	Insertion depth: 373° Partial insertion, two contacts outside Location: Scala tympani Scala deviation: No Tip fold-over: No
05_Left	Diameter (A) = 9.3 mm Width (B) = 6.9 mm Height (H) = 3.5 mm CDL (from RW) = 33.8 mm	Insertion depth: 475° Full insertion Location: Scala tympani Scala deviation: No Tip fold-over: No
05_Right	Diameter (A) = 9.3 mm Width (B) = 7.1 mm Height (H) = 3.7 mm CDL (from RW) = 34.7 mm	Insertion depth: 542° Full insertion Location: Scala tympani Scala deviation: No Tip fold-over: No
06_Left	Diameter (A) = 9.0 mm Width (B) = 6.4 mm Height (H) = 4.0 mm CDL (from RW) = 31.6 mm	Insertion depth: 483° Partial insertion, two contacts outside Location: Scala tympani
		Scala deviation: No Tip fold-over: No
06_Right	Diameter (A) = 8.8 mm Width (B) = 6.5 mm Height (H) = 3.5 mm CDL (from RW) = 31.5 mm	Insertion depth: 645° Full insertion Location: Scala tympani Scala deviation: No Tip fold-over: No
07_Left	Diameter (A) = 8.4 mm Width (B) = 6.3 mm Height (H) = 3.5 mm CDL (from RW) = 30.4 mm	Insertion depth: 570° Full insertion Location: Scala tympani Scala deviation: No Tip fold-over: No
07_Right	Diameter (A) = 9.3 mm Width (B) = 6.8 mm Height (H) = 3.7 mm CDL (from RW) = 33.5 mm	Insertion depth: 377° Partial insertion, one contact outside Location: Scala tympani Scala deviation: No Tip fold-over: No

## Discussion

In this study, the quality of the electrode insertion through a robotically drilled direct tunnel was validated using endoscopic and fluoroscopic supervision. To our knowledge, this is the first report of an electrode insertion study conducted using a robotic approach under fluoroscopic supervision. In 12 out of 15 analyzed cases, it was possible to fully insert the electrode and in all the cases, a steady electrode insertion was observed with no sign of scala deviation or tip-fold-over. This lack of complications may be due to feedback from fluoroscopic supervision, a high level of surgical CI experience among the participating surgeons, and the fact that this was an experimental setting. The three partial insertion cases could be due to formalin fixation of the specimens and hence increased endolymph viscosity in scala tympani. Without the mastoidectomy, the otomicroscopic visual feedback to the surgeon is lost and therefore endoscopic supervision was obtained through the tympanomeatal flap. However, creating the tympanomeatal flap implies an additional surgical procedure performed on the patient. Furthermore, manual insertion of the electrode array while simultaneously holding an endoscope can be a demanding task for the surgeon, hence influencing the placement of an electrode in the cochlea. However, due to the fixation of the head, an endoscope stand could help in this regard. A recent study (11) has shown the feasibility of a multiport approach through the HEARO procedure. This would imply the creation of a tunnel through the facial recess for electrode insertion and a second tunnel for placement of endoscope for visual inspection. By doing so, the endomeatal procedure could be avoided, enabling the surgeon to fit the endoscope in the holder within the tunnel. The future advancement toward robotic insertion of the electrode array would also facilitate a slower and more consistent electrode insertion.

The main purpose of our study was to gain access to the RW solely by the image-guided robotic system and to test the feasibility of the Robot HEARO surgery technique. The image-guided robot system with dedicated OTOPLAN software has shown to be highly precise and safe, considering the microanatomy with the closely adjacent facial nerve and chorda tympani as previously described by Bell (4), Caversaccio (2), and Weber (5). In live patients, there is the same concern regarding the safety of facial nerve and chorda tympani in the surgical approach for CI. In this respect, Postelmans et al. (12) compared mastoidectomy with posterior tympanotomi (MPTA) with suprameatal approach (SMA) and proposed the latter as a safe and effective technique. No reduction in post-operative complications was demonstrated and in addition, this approach showed to be more hazardous to ossicles, which is an additional obstacle in maintaining the level of residual hearing. The SMA technique includes an increased risk of electrode kinking as a consequence of the difficult 30° more superior insertion of the electrode. In a recent publication, Topsakal et al. (13) compared the surgical techniques including MPTA, SMA, and robotic techniques for cochlear implantation in terms of the trajectories toward the inner ear. They concluded that posterior tympanotomi (PT) approaches allow much smaller angles of the cochlear approach (ACA) than those for SMA. They have also found that within different PT modalities, robotically assisted surgery provides the most optimal ACA, which is the prerequisite for easy access to an array and the best possible placement of the electrode in the cochlea. The most optimal ACA is vital for optimal positioning of the electrode array and residual hearing preservation in CI surgery. We believe that the Robot HEARO surgery technique together with the future robotic insertion of the electrode will increase the precision and standardize the CI surgery.

Finally, in this study, fluoroscopic supervision of the electrode insertion was also performed to visualize the trajectory and advancement of the array inside the cochlea. The fluoroscopic supervision has previously been found very useful on patients when combined with electrophysiological measurements study (14). Fluoroscopic monitoring is mainly used in research and not in mainstream CI surgery in our Clinic. Radiation exposure during this procedure is very low and was never a limiting factor in human studies. The use of human cadavers allowed us to monitor the different stages with CBCT, without considering accumulated radiation to the patient. In future clinical studies, we will have to consider the radiation exposure to the patient and reduce the use of intra-operative CBCT scans, based on the data of our cadaver study. Furthermore, if the technique becomes an established surgical procedure in daily clinical practice, the results from the radiology taken at different stages of the surgery during both the cadaver and the clinical studies will provide important information on which stages radiology is in excess of and can be avoided.

## Conclusions

Electrode insertion with the robotic middle and inner ear access with the HEARO procedure is validated and found to be feasible and safe. It is a further step toward clinical application. The next stage of this study would be to perform the same methodology in clinical practice.

## Data Availability Statement

The raw data supporting the conclusions of this article will be made available by the authors, without undue reservation.

## Ethics Statement

The Regional Committee for Medical and Health Research Ethics of Northern Norway (REC North, reference: 2018/378/REK nord) evaluated the study. Since the study was conducted on tissue from deceased persons and therefore not considered as research on humans in accordance with the Norwegian Research Health Act, the Ethics Committee concluded that the study was outside of their mandate. Thus, the study has been planned and conducted in accordance with the research policy and routines of Oslo University Hospital.

## Author Contributions

GJ: concept, design, and writing. GJ and TS: supervision, resource, and literature search. GJ, BF-J, and TS: materials, data collection, and processing. GJ, BF-J, TS, MB, MI, RG, LO, HK, and MM: analysis, interpretation, and critical reviews. All authors contributed to the article and approved the submitted version.

## Funding

The study was funded by Oslo University Hospital.

## Conflict of Interest

The authors declare that the research was conducted in the absence of any commercial or financial relationships that could be construed as a potential conflict of interest. The study was conducted with service and collaboration from MED-EL, Innsbruck, Austria, and CASCINATION, Bern, Switzerland.

## Publisher's Note

All claims expressed in this article are solely those of the authors and do not necessarily represent those of their affiliated organizations, or those of the publisher, the editors and the reviewers. Any product that may be evaluated in this article, or claim that may be made by its manufacturer, is not guaranteed or endorsed by the publisher.

## Supplementary Material

Video 1 RW electrode insertion.

Video 2 Fluoroscopy of electrode insertion.

Video 3 The milling of the inner ear access.
